# Case Report: Fatal presumptive idiosyncratic hepatic necrosis secondary to standard-dose leflunomide administration in a dog

**DOI:** 10.3389/fvets.2025.1529324

**Published:** 2025-05-07

**Authors:** Cristina Aponte-Colon, Lauren A. Cochran, Lauren Harris, Erick A. Mears, Steven W. Frederick

**Affiliations:** ^1^Blue Pearl Veterinary Partners, Tampa, FL, United States; ^2^Blue Pearl Veterinary Science, Atlanta, GA, United States

**Keywords:** case report, immune-mediated, polyarthritis, IMPA, liver enzyme

## Abstract

An 8-year-old neutered male Greyhound was presented with intermittent lameness and joint pain, leading to a diagnosis of idiopathic immune-mediated polyarthritis. The initial treatment regimen involved prednisone and minocycline. Leflunomide (3 mg/kg PO q24h) was prescribed later for secondary immune modulation. Five weeks later, the dog exhibited signs of acute lethargy, weakness, and increased liver enzyme activities (alanine aminotransferase [ALT] 6,613 U/L and aspartate aminotransferase [AST] 3,718 U/L). Despitereceiving supportive care, the dog died. Necropsy revealed massive hepatic necrosis with effaced hepatic architecture and centrilobular pools of free blood, similar to findings noted in people with leflunomide-induced hepatic injury. This case highlights a previously unreported severe, idiosyncratic hepatotoxicity associated with standard doses of leflunomide in dogs, illustrating the need for vigilant monitoring and increased awareness of the potential need for dose adjustment if liver enzyme elevations occur.

## Introduction

Leflunomide is an immunomodulatory drug that is increasingly used in veterinary medicine to manage immune-mediated diseases, particularly those that are resistant to conventional therapies (1). Leflunomide’s mechanism of action involves inhibiting pyrimidine synthesis, which reduces lymphocyte proliferation and activity (2). Common adverse effects in dogs include gastrointestinal issues and myelosuppression (3). In humans, leflunomide can lead to hepatotoxicity, varying from mild liver enzyme elevations to severe liver injury (4). Reports of hepatotoxicity in dogs are primarily limited to mild, transient liver enzyme elevations, with no severe hepatotoxicity reported (5). This case report presents a rare and severe occurrence of presumptive leflunomide-induced hepatic necrosis in a dog, highlighting a potential side effect of this medication and the significance of routine liver enzyme monitoring during therapy.

## Case report

An 8-year-old, 32.9-kg, neutered male Greyhound was presented to a primary care veterinarian for outpatient evaluation of intermittent lameness and joint pain. During the physical examination, the dog was limping on the left hindlimb and showed pain on palpation of the neck and flexion of the right shoulder. No apparent joint effusion was observed. The dog exhibited a 2-year history of inflammatory bowel disease, which was diagnosed through endoscopic biopsies and effectively managed with a novel protein diet. A complete blood count (CBC), serum biochemistry panel, tick serology, and polymerase chain reaction (PCR) assay (*Anaplasma phagocytophilum*, *Anaplasma platys*, *Babesia canis, Babesia* sp. (*Coco*), *Babesia conradae*, and *Babesia gibsoni*), (*Bartonella henselae*, *Bartonella vinsonii*, *Ehrlichia canis*, *Ehrlichia chaffeensis*, *Ehrlichia ewingii*, *Mycoplasma haemocanis*, *Cand. M. haematoparuvm*, *Neorickettsia risticii*, and *Rickettsia rickettsii*), and orthogonal thoracic radiographs were conducted and the findings were largely unremarkable. The dog was prescribed minocycline (TEVA Pharmaceuticals, Sellersville, PA, United States; 6 mg/kg PO q12h) for 21 days and prednisone (HIMKA Pharmaceuticals, Amman, Jordan; 1.2 mg/kg PO q12h) due to initial concern for a possible rickettsial infection. However, diagnostic testing for tick-borne diseases ultimately turned negative, leading to the discontinuation of the antibiotic therapy.

Six weeks after the initial evaluation, the dog was presented to a veterinary referral practice for lethargy and hyperthermia. On presentation, the dog was calm but aware, ambulatory, and well-hydrated. Rectal temperature was slightly elevated at 39.3°C (102.7°F). Physical examination was unremarkable except for subjective splenomegaly on abdominal palpation and discomfort experienced when extending the right coxofemoral joint. CBC and serum biochemistry indicating a leukocytosis (white blood cell count [WBC]: 18.52–10^9^/L, reference interval [RI]: 5.05–16.76 × 10^9^/L) with a mild neutrophilia (15.73 × 10^9^/L, RI: 2.95–11.64 × 10^9^/L) but were otherwise unremarkable, including alanine aminotransferase (ALT) levels (52 U/L, RI: 12–118 U/L). Three-view thoracic radiographs were obtained unremarkable. The abdominal ultrasound indicated diffuse splenomegaly, characterized by normal architecture. Furthermore, the ultrasound-guided cytology of the spleen demonstrated the presence of typical splenic cells and stroma. The remainder of the abdominal ultrasound was unremarkable. A surgical consultation indicated right-sided coxofemoral joint pain during extension. The orthogonal radiographs of the pelvic limb revealed normal pelvic anatomy, although mild bilateral stifle effusion was noted. Bilateral stifle arthrocentesis was performed without complications. The fluid exhibited high cellularity (nucleated cell count 40 × 10^9^/L, RI: 2.5–3 × 10^9^/L), consisting of 91% non-degenerate neutrophils and fewer macrophages surrounded by a backdrop of pink-stained protein. No microorganisms were observed. Findings were primarily consistent with an inflammatory arthropathy, which is presumed to be idiopathic immune-mediated polyarthritis (IMPA). Aerobic and anaerobic bacterial cultures of synovial fluid from the left and right stifles showed no bacterial growth and the repeat tick disease PCR was also negative.

**Figure 1:**
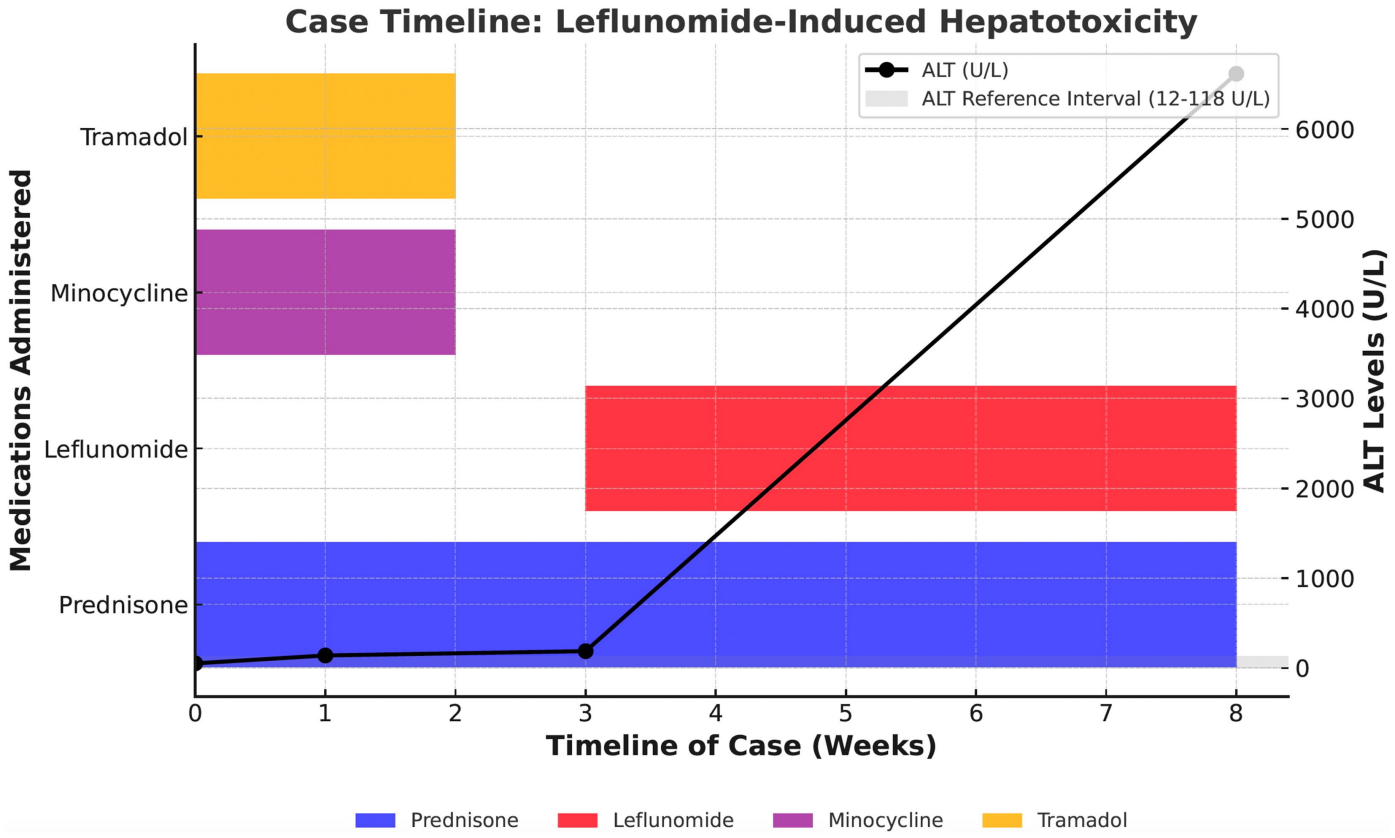
Case timeline of leflunomide-induced hepatotoxicity in an 8-year-old neutered male Greyhound. This figure illustrates the administration of prednisone, leflunomide, minocycline, and tramadol over an 8-week period, along with trends in ALT levels (in U/L). The ALT reference interval (12–118 U/L) is represented by the shaded gray area. A progressive increase in ALT is observed, with a marked elevation following leflunomide administration. This figure highlights the temporal relationship between medication administration and hepatocellular enzyme elevations, suggesting a potential association between leflunomide and severe hepatotoxicity.

The dog was hospitalized following arthrocentesis prior to discharge. Medical therapies prescribed for presumed idiopathic IMPA included tramadol (Amneal Pharmaceuticals, Glasgow, KY, United States; 3 mg/kg PO q8h), minocycline (6 mg/kg PO q12h), prednisone (0.6 mg/kg q12h), and leflunomide (TEVA Pharmaceuticals, Sellersville, PA, United States; 3 mg/kg PO q24h). Tramadol and minocycline were administered empirically for 3 weeks due to concerns about a rickettsial infection but were discontinued 5 weeks prior to the onset of acute hepatic injury (Figure 1).

Leflunomide was added as a secondary immune modulatory agent due to the dog’s previous significant muscle loss and weakness associated with high-dose prednisone prescribed at the initial onset of intermittent lameness. The dog initially responded very well to treatment with resolution of lethargy, fever, and joint effusion within 10 days of initiating the medication regimen. Serial therapeutic monitoring using serum biochemistry and CBC was performed 10 and 21 days after initiating the medications, revealing a progressive mild elevation of ALT (ALT – day 10: 140 U/L, day 21: 188 U/L, RI: 10–118 U/L), but was otherwise unremarkable. During both evaluations, the dog was normothermic and showed progressive weight gain with no apparent joint effusion or lameness observed. No changes to the treatment plan were recommended.

Eight weeks after starting treatment for IMPA, the dog was evaluated by the primary care veterinarian for acute onset lethargy that progressed to weakness and recumbency in less than 24-h. On examination, the dog was normothermic but showed weak-to-dull mentation, cold extremities, muddy mucous membranes, and prolonged capillary refill time. Serum biochemistry and CBC revealed a leukopenia (2.62 × 10^9^/L, RI: 6.0–17.0 × 10^9^/L) characterized by a lymphopenia (1.0 × 10^9^/L, RI: 1.0–4.8 × 10^9^/L), thrombocytopenia (81 × 10^9^/L, RI: 120–600 × 10^9^/L), increase in liver enzyme activity (ALT – 6,613 U/L, RI: 3–50 U/L), AST (3,718 U/L, RI: 1–37 U/L), alkaline phosphatase (ALP – 673 U/L, RI: 20–155 U/L), and hyperbilirubinemia (total bilirubin 0.8 mg/dL, RI: 0.1–0.7 mg/dL). Coagulation testing was not performed. Hospitalization and supportive care were recommended; however, attempts at peripheral intravenous catheterization were not successful. The dog received subcutaneous fluids (30 mg/kg Lactated Ringer’s solution, B. Braun Medical Inc.) and was discharged with plans to return the next day for care. Within 1 h of returning home, the dog died suddenly.

The dog’s remains were sent for necropsy at Colorado State University’s College of Veterinary Medicine, where pathologic evaluation was performed by a board-certified veterinary pathologist. Severe-to-moderate necrosis of the liver was observed, mainly focused on the central veins. The hepatic architecture was distorted, showing no viable hepatocytes or remnants of the cord architecture in more than half of the liver parenchyma. Vacuolar change was also noted in hepatocytes adjacent to areas of hepatocellular necrosis. The centrilobular region contained pools of free blood. Evidence of chronic edema with low protein fluid was present in the lungs along with the acute formation of fibrin thrombi in small pulmonary blood vessels. In the heart, aggregates of mild multifocal inflammation were dispersed throughout the myocardium. The kidneys exhibited terminal congestion of the capillaries along with finely granular calcified deposits in Bowman’s capsules. The stomach and small intestines exhibited multiple areas of chronic inflammation, characterized by varying degrees of lymphocytes and plasma cells, from mild to severe. The remaining organs were histologically normal. The lesions identified in the liver were deemed characteristic of acute toxic hepatic injury. It was determined that the cause of immediate death was massive hepatic necrosis, which is consistent with known outcomes of severe toxic hepatic injury resulting in acute hepatic failure and death (6).

## Discussion

This case report outlines fulminant, presumptive idiosyncratic hepatic necrosis in a dog receiving a standard dose of leflunomide. To date, only mild hepatotoxicity, defined as mild elevations in liver enzyme activity, has been reported in up to 6.3% of dogs receiving leflunomide for immune-mediated diseases. Increased liver enzyme activity typically occurs within 2 weeks of starting treatment initiation and has been reported to resolve over several weeks with a 50% dose reduction (5). Leflunomide has also been identified as a potential risk factor for hepatotoxicity in miniature dachshunds (7).

Leflunomide-induced liver injury in humans has been reported in as many as 16% of cases, with rare instances of acute liver failure that require liver transplantation or lead to death (8–11). Histopathological findings in affected humans may include centrilobular hepatic necrosis, portal and periportal inflammation, periportal fibrosis, and steatosis (9–11). The necropsy findings of the dog included centrilobular necrosis, periportal inflammation, and moderate steatosis, which are consistent with findings observed in humans with leflunomide-induced hepatic injury; however, histopathologic images were unavailable due to the laboratory’s image retention protocols. These findings are consistent with the World Small Animal Veterinary Association’s (WSAVA) criteria for severe hepatocellular damage. This includes centrilobular to panlobular necrosis, marked hepatocellular swelling, and inflammation primarily located in the portal and periportal regions (11).

Leflunomide-associated hepatotoxicity is considered idiosyncratic, indicating that it is an unpredictable off-target effect that does not coincide with the drug’s primary mechanism of action (12). Hepatotoxicity has been connected to specific cytochrome P450 genetic variants, namely *CYP 2C92 and CYP 2C9*3, which are known to impair the normal metabolism of the drug (13, 14). This impaired metabolism can result in the accumulation of reactive metabolites, leading to oxidative stress, mitochondrial dysfunction, and the formation of reactive oxygen species, which can ultimately cause hepatic failure (13, 15–17). Centrilobular hepatocytes have higher concentrations of cytochrome P450 enzymes and are more susceptible to this particular type of injury.

Although there was no evidence of underlying liver disease on serum biochemistry or abdominal ultrasound prior to leflunomide initiation, both preprandial and postprandial serum bile acids were not assessed either before or during leflunomide therapy. While bile acids are often used as a marker of hepatic functional reserve, their diagnostic utility in acute hepatic necrosis is variable. In severe acute hepatocellular necrosis, liver enzyme increases frequently precede significant changes in bile acid concentrations, as synthetic function may be preserved until more advanced stages of hepatic failure (18, 19). Furthermore, the development of hypoxic centrilobular necrosis can impair hepatic clearance without necessarily leading to an immediate, marked increase in circulating bile acids (14). The total bilirubin levels in our dog were mildly elevated at 0.8 mg/dL (RI: 0.1–0.7 mg/dL) at the time of death, which appears discordant with the extensive panlobular necrosis observed histologically, possibly reflecting an idiosyncratic reaction or variation in bilirubin metabolism.

In humans, monthly serum liver enzyme evaluation is recommended during the first 6 months of leflunomide therapy. Minor elevations in ALT or AST (up to twice the reference interval maximum) prompt monitoring of liver values in 2 weeks and consideration for leflunomide dose reduction. A dose reduction is recommended if moderate elevations in liver aminotransferases are noted (greater than twice the reference interval maximum). If moderate increases in aminotransferases continue despite dose reduction, withdrawal of the drug is recommended (20). In this case, the dog’s liver enzyme activities were mildly elevated at two serial evaluations within 3 weeks of initiating leflunomide therapy. Still, they were not re-evaluated until the dog was presented with acute disease 5 weeks later. Despite adhering to a monitoring schedule similar to human guidelines, the dog in this report experienced an acute and progressive hepatopathy. This illustrates the need for vigilant monitoring and heightened awareness of dose adjustments should liver enzyme elevations be encountered. Given the progressive nature of the hepatopathy observed, monitoring liver enzyme activity every 2 weeks during the first 2 months of treatment may be warranted in dogs receiving leflunomide, mainly when mild elevations are detected.

Leflunomide in humans has also been associated with drug-induced autoimmune phenomena, including drug-induced lupus and immune-mediated hematologic disorders such as pancytopenia (17). As such, routine monitoring of hematologic patterns and autoimmune markers such as antinuclear antibody (ANA) is often recommended in human patients receiving long-term therapy (21). While similar autoimmune complications have not been extensively reported in veterinary medicine, consideration of periodic autoimmune panel testing in dogs receiving chronic leflunomide therapy may be prudent, particularly in cases with unexplained systemic signs or cytopenias.

Both chronic administration of tramadol and concurrent administration of leflunomide and minocycline have both been described as risk factors for hepatotoxicity (10, 22). The dog in this case received both tramadol and minocycline early in the diagnostic process. Yet, it is unlikely that either tramadol or minocycline contributed to the dog’s hepatotoxicity due to the short half-lives of 1.7 h and 4–7.3 h for tramadol and minocycline, respectively (14, 23). Additionally, histopathologic findings in this dog were inconsistent with tramadol-associated hepatic injury (hepatocellular degeneration and necrosis), further supporting that tramadol was not a primary contributor. Minocycline-induced liver injury in humans typically manifests within 1–3 months of initiating therapy, often with an immune-mediated pattern of hepatotoxicity, including periportal inflammation, necroinflammatory activity, or granulomatous hepatitis (24). These features are inconsistent with the acute and severe hepatic necrosis observed in this case.

Necropsy findings suggest that the dog in our case died from complications of acute hepatic necrosis and acute hepatic failure within 2 months of initiating leflunomide and likely secondary multiorgan dysfunction. When the dog presented in acute decline, testing for coagulopathy and encephalopathy was not performed; however, at necropsy, pools of free blood were identified in the central area of hepatic lobules, which may indicate coagulopathy. Leflunomide-induced hepatotoxicity in humans has been associated with coagulopathy secondary to hepatic synthetic dysfunction, which may manifest as prolonged prothrombin time (PT) and activated partial thromboplastin time (aPTT) (12, 18). Along with routine liver enzyme and CBC monitoring, coagulation testing may be indicated if hepatic enzyme elevations occur or if clinical signs of coagulopathy develop. Likewise, though a fasting ammonia level was not performed, the authors suggest that hepatic encephalopathy could have contributed to the acute (<24 h) progression from lethargy to weakness and recumbency.

In summary, we describe the first case of presumptive idiosyncratic hepatic necrosis secondary to standard dose leflunomide administration in a dog. The potential for this severe adverse effect should be considered when leflunomide is prescribed. Periodic monitoring of liver enzyme activity is likely warranted during the first 6 months of leflunomide therapy, emphasizing recognizing even subtle elevations. If new or increased liver enzyme elevations are documented during the initial stages of leflunomide therapy, a dose reduction or discontinuation of leflunomide may be prudent.

## Data Availability

The original contributions presented in the study are included in the article/supplementary material, further inquiries can be directed to the corresponding author.
